# Distinct behavior in early life stress dams predicts heterogeneity in future stress response in offspring over lifespan

**DOI:** 10.1192/j.eurpsy.2022.253

**Published:** 2022-09-01

**Authors:** F. Mey, G. Treccani, U. Schmitt, M. Müller

**Affiliations:** 1 AG Translationale Psychiatrie, Psychiatrie Und Psychosomatik, Mainz, Germany; 2 Institut für Mikroskopische Anatomie und Neurobiologie, Anatomie, Mainz, Germany; 3 Leibniz-Institut für Resilienzforschung (LIR) gGmbH, Ag Müller, Mainz, Germany

**Keywords:** chronic social defeat, mice experiment, observerindependent behavioral characterisation, early life stress

## Abstract

**Introduction:**

Exposure to early life stress (ELS) strongly predicts prevalent, impairing, and costly psychiatric illness throughout life including mental disorders. The reason, some individuals are more vulnerable to ELS whereas others remain resilient, is poorly understood. There is a need for better understanding of early biological changes triggered by ELS with responsibility to negative outcomes in health.

**Objectives:**

We stratify animals after ELS according to corticosterone levels. [1] Re-challenging the animals to a second stressor, chronic social defeat (CSD) [2], in adulthood was performed to understand individual trajectories depending on corticosterone exposure during early adverse conditions.

**Methods:**

We performed ELS as previously reported [1]. Behavior of mothers was observed during ELS. Correlation between level of corticosterone and behavior observed in dams. ELS animals were exposed to a second stress in adulthood. A battery of tests for different behavioral domains was performed. Behavioral analyses was combined with assessment of litter HPA system reactivity and observed behavior in dams.

**Results:**

Stress dams where significantly higher in number of sorties over whole observation period, time dams spent outside the nest differed. We could correlate the number of sorties on p3 with corticosterone plasma level at p9. Control dams spent significantly more time outside in 9pm recordings than stress animals. We could show reduced interaction with social juvenile targets in sociability test for CSD mice. Light dark transition was significantly higher for control mice compared to CSD but lower for control vs ELS animals.

**Conclusions:**

Behavior in dams during ELS correlates with chronic stress coping mechanisms in offspring’s adulthood.

**Disclosure:**

No significant relationships.

